# Associations between new and old anthropometric indices with type 2 diabetes mellitus and risk of metabolic complications: a cross-sectional analytical study

**DOI:** 10.1590/1677-5449.200236

**Published:** 2021-09-20

**Authors:** Parichehr Amiri, Ahmad Zare Javid, Leila Moradi, Neda Haghighat, Rahim Moradi, Hossein Bavi Behbahani, Milad Zarrin, Hadi Bazyar

**Affiliations:** 1 Ahvaz Jundishapur University of Medical Sciences, Student Research Committee, Ahvaz, Iran.; 2 Ahvaz Jundishapur University of Medical Sciences, School of Allied Medical Sciences, Department of Nutrition, Ahvaz, Iran.; 3 Ahvaz Jundishapur University of Medical Sciences, Health Research Institute, Diabetes Research Center, Ahvaz, Iran.; 4 Shiraz University of Medical Sciences, Laparoscopy Research Center, Shiraz, Iran.; 5 Ahvaz Jundishapur University of Medical Sciences, School of Medicine, Department of Clinical Biochemistry, Ahvaz, Iran.

**Keywords:** anthropometry, obesity, type 2 diabetes mellitus, measures of association, exposure, risk or outcome, antropometria, obesidade, diabetes melito tipo 2, medidas de associação, exposição, risco ou resultado

## Abstract

**Background:**

Obesity can increase the risk of diabetes mellitus and complications associated with it.

**Objectives:**

The aim of this study was to estimate the associations between new and old anthropometric indices and the risk of type 2 diabetes mellitus (T2DM) and its metabolic complications.

**Methods:**

In this cross-sectional analytical study, 110 T2DM subjects and 110 healthy controls were selected by convenience sampling. Metabolic factors were evaluated including the atherogenic index of plasma (AIP), glycemic status, lipid profile, blood pressure, kidney indices, new anthropometric indices (abdominal volume index [AVI], body shape index [ABSI], lipid accumulation product [LAP], body adiposity index [BAI], and conicity index [CI]), and old anthropometric indices (weight, body mass index [BMI], and waist and hip circumference [WC and HC]).

**Results:**

Significant positive correlations were observed between AVI, LAP, and BAI and fasting blood glucose and HbA1c in the T2DM group (p < 0.001 for all associations). The odds ratio (OR) for T2DM elevated significantly with increasing BMI (OR: 1.30, 95% CI: 1.20-1.42), LAP (OR: 1.20, 95% CI: 1.13-1.27), and BAI (OR: 1.32, 95% CI: 1.21-1.43). The indices AVI (OR: 1.90, 95% CI: 1.57-2.29), LAP (OR: 1.19, 95% CI: 1.13-1.27), BAI (OR: 1.19, 95% CI: 1.12-1.26), WC (OR: 1.29, 95% CI: 1.18, 1.42), and HC (OR: 1.07, 95% CI: 1.01, 1.14) significantly increased the risk of metabolic syndrome (MetS).

**Conclusions:**

Associations were identified between obesity indices and diabetes. These indices could be used in clinical practice for evaluation and control of T2DM.

## Resumo

**Contexto:**

A obesidade pode aumentar o risco de diabetes melito e complicações associadas.

**Objetivos:**

O objetivo deste estudo foi estimar a associação de índices antropométricos novos e antigos com o risco de diabetes melito tipo 2 (DM2) e suas complicações metabólicas.

**Métodos:**

Neste estudo analítico transversal, 110 indivíduos com DM2 e 110 controles saudáveis foram selecionados por amostragem de conveniência. Foram avaliados os fatores metabólicos, incluindo índice aterogênico plasmático, estado glicêmico, perfil lipídico, pressão arterial, índices renais, índices antropométricos novos [índice de volume abdominal (AVI), índice de formato corporal (ABSI), produto de acumulação lipídica (LAP), índice de adiposidade corporal (BAI) e índice de conicidade (CI)] e índices antropométricos antigos [peso, índice de massa corporal (IMC), circunferência de cintura e quadril].

**Resultados:**

Foi observada uma correlação positiva significativa de AVI, LAP e BAI com glicemia de jejum e hemoglobina glicada no grupo DM2 (p para todos < 0,001). A *odds ratio* (OR) do grupo DM2 foi significativamente elevada com aumento de IMC [OR: 1,30, intervalo de confiança (IC) de 95%: 1,20-1,42], LAP (OR: 1,20, IC95%: 1,13-1,27) e BAI (OR: 1,32, IC95%: 1,21-1,43). Os índices AVI (OR: 1,90, IC95%: 1,57-2,29), LAP (OR: 1,19, IC95%: 1,13-1,27), BAI (OR: 1,19, IC95%: 1,12-1,26), WC (OR: 1,29, IC95%: 1,18-1,42) e HC (OR: 1,07, IC95%: 1,01-1,14) aumentaram significativamente o risco de síndrome metabólica.

**Conclusões:**

Foi reconhecida uma associação entre índices de obesidade e diabetes. Esses índices podem ser usados na prática clínica para avaliação e controle do DM2.

## INTRODUCTION

Type 2 diabetes mellitus (T2DM) is a chronic disease characterized by abnormal blood glucose levels. It mainly develops when insulin secretion or action are impaired.^1^ The International Diabetes Federation (IDF) stated that 1 in 11 adults (415 million) were diabetic in 2015. According to the IDF’s estimate, the global prevalence of diabetes mellitus (DM) may be more than 642 million people by 2040.^2^

DM and its complications are crucial health concerns which should be considered. Most DM patients experience at least one complication.^3^ Complications can be categorized as microvascular (nephropathy, retinopathy, and neuropathy) or macrovascular (peripheral vascular disease, coronary artery disease, and cerebrovascular disease). T2DM usually occurs within the context of the metabolic syndrome (MetS), which is a cluster of conditions including abdominal obesity, hyperlipidemia, and hypertension. These factors can also act to promote macrovascular complications. All complications are responsible for DM-related morbidity and mortality.^4^

A complicated unknown interaction between environmental agents (sedentary life style, unhealthy diets, stress, obesity, etc.) and genetic factors (ethnic and racial differences, family history, etc.) contributes to the onset and progression of T2DM.^5^^-^ 7 On the other hand, the prevalence rates of obesity and T2DM are increasing simultaneously, which suggest that presence of obesity is a major risk factor for T2DM.^8^ Based on prior studies, obese subjects have altered levels of hormones, proinflammatory cytokines, non-esterified fatty acids, and other elements which are associated with insulin resistance and can lead to development of T2DM.^9^ In particular, studies indicate that there is a strong relationship between abdominal or visceral obesity and metabolic abnormalities.^10^ The risk of developing T2DM is 3 to 5 times greater in patients with MetS.^11^ Therefore, it is essential to assess the relationship between anthropometric indices and DM risk.

There are several anthropometric methods for measuring central and general obesity. As a common indicator of general obesity, body mass index (BMI) is identified as an important risk factor for T2DM. Indicators of abdominal obesity such as waist circumference (WC) and waist-to-hip ratio (WHR) are also considered as predictors of T2DM risk, independent of BMI.^12^ However, these traditional anthropometric measures do not discriminate visceral and subcutaneous fat.^13^ Furthermore, there are no ideal measurements to evaluate obesity, particularly when used to assess disease risk.

New anthropometric indices have been recently proposed that are easily applicable, low cost, and able to identify body fat distribution. These new anthropometric indices include a body shape index (ABSI), the adiposity volume index (AVI), the body adiposity index (BAI), the lipid accumulation product index (LAP), and the conicity index (CI).^14^ However, the correlations between these new indices and risk of T2DM and metabolic disturbances have not been completely studied. The ability to evaluate patients at risk is essential to ensure implementation of the most appropriate treatment strategies. The aim of the present study was to evaluate the associations between new and old anthropometric indices and risk of T2DM and of its metabolic complications.

## METHODS

### Participants

This cross-sectional analytical study was conducted from February 2019 to September 2019. In this study, 120 T2DM patients who had been referred to the Endocrinology and Metabolism clinic at Golestan Hospital, Ahvaz Jundishapur University of Medical Science, were enrolled on the study based on inclusion criteria (willingness to participate in the study, male and females with DM for more than 5 years, aged between 18 and 60 years) using a convenience sampling method. Lactating and pregnant women, smokers, alcohol consumers, patients with insulin injection, patients with other chronic diseases, and patients using antibiotics, immunosuppressive medications, or dietary supplements were excluded from the study. Also, 120 healthy controls (matched for sex and age and free from DM) were chosen from the same center using a convenience sampling method simultaneously with the case group. Exclusion criteria for the control group were similar to those for the case group.

### Calculation of Metabolic Syndrome (MetS)

The prevalence of MetS was determined among the population in our study. According to the International Diabetes Federation (IDF) criteria for the Iranian population, MetS was defined as existence of central adiposity or waist circumference (WC) ≥95 cm for both genders (according to the Iranian National Committee of Obesity);^15^ plus two or more of the following four factors: high serum triglyceride concentration (≥150 mg/dL); low serum high-density lipoprotein cholesterol (HDL-c) (<50 mg/dL for women and <40 mg/dL for men); high fasting blood sugar (FBS) (≥100 mg/dL), and high blood pressure (≥130/85 mm Hg).^16^^,^ 17

### Ethics approval

Written informed consent was obtained from all participants at the beginning of the study. The research protocol was in accordance with the Declaration of Helsinki guidelines. The ethics committee at Ahvaz Jundishapur University of Medical Sciences approved the study (Ethical Code: IR.AJUMS.REC.1397.906).

### Measurement of new and old anthropometric indices

Height, weight, WC, and hip circumference (HC) were measured by a trained nutritionist. A Seca scale was used for measuring weight (light clothing without shoes, precision of 0.5 kg). Height was measured with a wall-mounted meter (precision of 0.1 cm). WC (the distance between the iliac crest and the rib at the end of exhalation), and HC (at the level of the widest circumference over the greater trochanters) were measured using a non-elastic tape.

The anthropometric indices were calculated using the following equations (Equation 1-8).^13^^,^ 18

$$\text{Body mass index }\left(\text{BMI}\right)=\frac{\text{weight}\left(\text{kg}\right)}{{\text{height}}^{2}}$$ (1)

$$\text{Waist}-\text{to}-\text{hip ratio }\left(\text{WHR}\right)=\frac{\text{WC}\left(\text{cm}\right)}{\text{HC}\left(\text{cm}\right)}$$ (2)

$$\text{A body shape index }\left(\text{ABSI}\right)=\frac{\text{wc}}{height^{1/2}\times {\text{BMI}}^{2/3}}$$ (3)

$$\text{Abdominal volume index }\left(\text{AVI}\right)=\left[2({\text{WC}}^{2}\right)+0.7\left(\text{waist}/\text{hip}{)}^{2}\right]/1000$$ (4)

$$\text{Body adiposity index}\;\left(\text{BAI}\right)=\frac{\text{hip}}{{\text{height}}^{1.5}}-18$$ (5)

$$\text{Conicity index }\left(\text{CI}\right)=\frac{\text{WC}\left(\text{m}\right)}{0.109\surd \frac{\text{weight}\left(\text{kg}\right)}{\text{height}\left(\text{m}\right)}}$$ (6)

$$\text{LAP for men}=\left[\text{WC}\left(\text{cm}\right)-65\right]\times \left[\text{triglyceride}\left(\text{mM}\right)\right]$$ (7)

$$\text{LAP for women}=\left[\text{WC}\left(\text{cm}\right)-58\right]\times \left[\text{triglyceride}\left(\text{mM}\right)\right]$$ (8)

Measurement of mean arterial pressure (MAP), pulse pressure (PP), and physical activity (PA)

The international physical activity questionnaire (IPAQ) was used to evaluate physical activity (PA).^19^ Blood pressure was measured using a mercury sphygmomanometer after 10 minutes at rest. Pulse pressure (PP) and mean arterial pressure (MAP) were computed according to the following equations (Equations 9 and 10);^20^

$$\text{PP }\left(\text{mmHg}\right)=\;SBP\left(\text{mmHg}\right)\;-\;DBP\left(\text{mmHg}\right)$$

$$\text{MAP }\left(\text{mmHg}\right)=\left[\text{SBP}+\left(2\times \text{DBP}\right)\right]/3$$ (10)

### Biochemical measurements

A blood sample (5^cc^) was obtained after an overnight fast. Serum levels of metabolic parameters were assessed by an enzymatic method using standard kits (Pars Azmon Co. kit, Tehran, Iran). Hemoglobin A1C (HbA1c) levels were evaluated using high-performance liquid chromatography (HPLC). A urine sample was also collected over a 24-hour period. Micro-albuminuria was defined as levels of albumin ranging from 30 to 300 mg in a 24-hour urine collection.

Low density lipoprotein (LDL-c), very low density lipoprotein (VLDL-c), atherogenic index of plasma (AIP), and estimated glomerular filtration rate (eGFR) were calculated according to the following equations (Equations 11-14);^21^^,^ 22

$$LDL-c\;\left(mg/dL\right)\;=\;Total\;cholesterol\;\left(mg/dL\right)\;-\;Triglycerides\;\left(mg/dL\right)\;/5\;\left(VLDL-c\right)\;–\;HDL-c\;\left(mg/dL\right)$$ (11)

$$VLDL-c=Triglycerides\;\left(mg/dl\right)\;/\;5$$ (12)

$$\text{AIP}=\log\frac{\text{Triglycerides}}{\text{HDL}}$$ (13)

$$\begin{array}{l} eGFR\;\left(mL/min\;per\;1.73\;m^{2}\right)\;=\;186\;\times \;{\left(SerumCreatinine)\right)}^{-1.154}\times \\ {\left(Age\right)}^{-0.203}\times \;\left(0.742\;if\;female\right)\;\times \;\left(1.210\;if\;African-American\right) \end{array}$$ 14

^21^

### Sample size

Based on the study by Rahmanian et al., and considering blood pressure as the main variable (α = 0.05 and β = 0.1), the sample size was calculated as 120 people in each group.^23^

### Statistical analysis

In this study, new anthropometric indices (AVI, ABSI, LAP, BAI, and CI), and old anthropometric indices (weight, BMI, WC, and HC) were considered as independent variables and metabolic parameters (fasting blood glucose [FBG], glycosylated hemoglobin [HbA1C], triglycerides [TG], total cholesterol [CHOL], HDL-c, LDL-c, VLDL-c, eGFR, blood urea nitrogen [BUN], creatinine [Cr], SBP, DBP, MAP, PP, and AIP) and the risk of DM and MetS were considered as dependent variables. Also, metabolic syndrome, gender, and education were considered as categorical variables and metabolic parameters (FBG, HbA1C, TG, CHOL, HDL-c, LDL-c, VLDL-c, eGFR, BUN, Cr, SBP, DBP, MAP, PP, and AIP) were considered as continuous variables. Normality of data was checked with the Kolmogorov-Smirnov test. Parametric variables are expressed as mean ± standard deviation (SD). The *t* test for independent samples was applied to compare means (SD) of normally distributed parameters. Correlations between anthropometric indices and metabolic parameters were assessed using the Pearson correlation test. Odds ratios (ORs) and their 95% confidence intervals (CIs) were reported using Logistic regression tests. Data were analyzed using SPSS version 19 for Windows (PASW Statistics). P-values less than 0.05 were considered significant.

## RESULTS

### General characteristics

Ten people from each group were excluded from the study because of a lack of study conditions and unwillingness to participate. A total of 220 participants (110 T2DM patients and 110 healthy controls) were involved in this study. The mean ages of the participants in the T2DM and control groups were respectively 50.65 ± 5.31 and 51.62 ± 5.6 years. The general characteristics of participants are shown in Table1.

**Table 1:** Characteristics of the subjects in the healthy and T2DM groups.

**Variables**	**Healthy group**	**Diabetic group**	***P- value***
Age (y)	51.62 ± 5.6	50.65 ± 5.31	0.18
Gender			
Female (N) (%)	72 (49)	75 (51)	0.66 *
Male (N) (%)	38 (52.1)	35 (47.9)	
Education			
Illiterate – elementary (N) (%)	64 (43.8)	82 (56.2)	0.056*
Middle school (N) (%)	24 (60)	16 (40)	
High school (N) (%)	13 (59.1)	9 (40.9)	
College (N) (%)	9 (75)	3 (25)	
Total	110	110	
Weight (kg)	66.79 ± 7.91	75.94 ± 11.52	<0.001
Height (cm)	165.78 ± 8.83	164.18 ± 10.87	0.23
Body mass index (kg/m^2^)	24.45 ± 3.57	28.22 ± 3.82	<0.001
Waist circumference (cm)	73.81 ± 4.40	103.46 ± 8.73	<0.001
Hip circumference (cm)	95.27 ± 4.05	107.73 ± 8.83	<0.001
Waist-to-hip ratio	0.77 ± 0.04	0.96 ± 0.07	<0.001
Waist-to-height ratio	0.44 ± 0.03	0.63 ± 0.06	<0.001
Physical activity (MET-min/week)	961.12 ± 548.26	286.93 ± 154.77	<0.001
Fasting blood glucose (mg/dL)	87.69 ± 7.78	165.17 ± 31.39	<0.001
Hemoglobin A1c %	4.84 ± 0.46	8.34 ± 1.31	<0.001
Triglycerides (mg/dL)	156.86 ± 62.22	148.94 ± 38.29	0.25
Total cholesterol (mg/dl)	167.29 ± 64.89	159.37 ± 34.55	0.26
High-density lipoprotein cholesterol (mg/dl)	47.50 ± 10.43	42.55 ± 10.28	<0.001
Low-density lipoprotein cholesterol (mg/dl)	89.27 ± 38.37	98.18 ± 41.76	0.1
Very low-density lipoprotein cholesterol (mg/dl)	31.37 ± 12.44	29.78 ± 7.65	0.25
Low-density/high-density lipoprotein cholesterol	2.04 ± 1.07	2.45 ± 1.36	0.01
Atherogenic index of plasma	0.49 ± 0.22	0.54 ± 0.15	0.08
Estimated glomerular filtration rate (mL/min/1.73m^2^)	80.23 ± 16.56	79.62 ± 18.32	0.79
Blood urea nitrogen (mg/dL)	12.69 ± 2.23	13.34 ± 3.01	0.07
Creatinine (mg/dL)	0.80 ± 0.14	0.86 ± 0.15	0.006
Albuminuria (mg/24h)	5.60 ± 2.09	10.62 ± 4.29	<0.001
Systolic blood pressure (mmHg)	118.72 ± 9.96	125.36 ± 13.11	<0.001
Diastolic blood pressure (mmHg)	73.45 ± 7.34	76.18 ± 8.34	0.01
Mean arterial pressure (mmHg)	88.54 ± 7.41	92.57 ± 8.40	<0.001
Pulse pressure (mmHg)	45.27 ± 7.98	49.18 ± 12.20	0.005
A body shape index (m^11/6^ Kg^-2/3^)	0.036 ± 0.01	0.037 ± 0.01	0.35
Abdominal volume index	10.93 ± 1.29	21.56 ± 3.54	<0.001
Conicity index	1.06 ± 0.08	1.40 ± 0.15	<0.001
Lipid accumulation product	24.45 ± 12.69	73. 43 ± 26.25	<0.001
Body adiposity index (Kg/m^2^)	26.87 ± 4.01	33.59 ± 6.39	<0.001
Metabolic syndrome			
Yes	0 (0)	77 (70)	<0.001*
No	110 (100)	33 (30)	
Total	110 (100)	110 (100)	

Values are expressed as means ± SD. *P <0*.*05* was considered as significant using the independent *t* test for comparison between the two groups;

* *P <0.05* was considered as significant using the Chi-square test.

### Comparison of anthropometric indices and metabolic parameters between the T2DM and healthy groups

Weight, BMI, WHR, WC, HC, and waist**-**to-height ratio were significantly greater in the T2DM group than in the healthy group (p < 0.001 for all comparisons). The T2DM group had significantly lower physical activity compared with the healthy group (p < 0.001). Except for ABSI, the other anthropometric indices including AVI, CI, LAP, and BAI were significantly lower in the healthy group than the T2DM group (p < 0.001 for all comparisons). Also, HDL-c (p < 0.001) was significantly lower and the LDL-c to HDL-c ratio (p = 0.01) was significantly higher in T2DM patients than the healthy group. SBP (p < 0.001), DBP (p = 0.01), MAP (p < 0.001), and PP (p = 0.005) were significantly higher in the T2DM group compared with the healthy group. The metabolic parameters eGFR, creatinine, and BUN did not significantly differ between the two groups (p ≥ 0.05), whereas albuminuria was significantly higher in T2DM patients than in the healthy controls (p < 0.001) (Table 1).

### T2DM risk assessment according to new and old anthropometric indices

LAP and BAI significantly increased the risk of T2DM even after adjustment for confounding factors (sex, age, physical activity, education, and medications) (OR: 1.21, 95% CI: 1.06-1.37; p = 0.003 and OR: 1.33, 95% CI: 1.10-1.62; p = 0.003, respectively). AVI showed the greatest OR (OR: 28.38, 95% CI: 0.99-812.80) for T2DM, followed by BAI (OR: 1.32, 95% CI: 1.21-1.43) and LAP (OR: 1.20, 95% CI: 1.13-1.27). In addition, the risk of T2DM was related to increasing weight, HC, and BMI both before and after adjustment for variables including sex, age, physical activity, education, and medications. WC increased the risk of T2DM (OR: 2.95, 95% CI: 1.02-8.55), but there was no significant difference after adjusting for confounding variables (Table 2).

**Table 2:** Odds ratios (95% CI) for T2DM according to new and old anthropometric indices.

**Variables**	**Or (CI)**	**B**	* ***P- value***
Body adiposity index (Kg/m^2^)			
Model 1a	1.32 (1.21-1.43)	0.27	< 0.001
Model 2b	1.34 (1.23-1.46)	0.29	< 0.001
Model 3c Model 4d	1.31 (1.17-1.46) 1.33 (1.10-1.62)	0.27 0.29	< 0.001 0.003
Lipid accumulation product			
Model 1^a^	1.20 (1.13-1.27)	0.18	< 0.001
Model 2^b^	1.21 (1.13-1.28)	0.19	< 0.001
Model 3^c^	1.30 (1.15-1.46)	0.26	< 0.001
Model 4^d^	1.21 (1.06-1.37)	0.19	0.003
Abdominal volume index			
Model 1^a^	28.38 (0.99-812.80)	3.34	0.051
Model 2^b^	29.34 (0.91-937.89)	3.37	0.056
Model 3^c^	59.83 (0.43-8206.14)	4.09	0.10
Model 4^d^	54.09 (0.29-10003.22)	16.19	0.98
Weight			
Model 1^a^	1.10 (1.06-1.13)	0.09	< 0.001
Model 2^b^	1.10 (1.06-1.14)	0.09	< 0.001
Model 3^c^	1.08 (1.03-1.13)	0.08	< 0.001
Model 4^d^	1.16 (1.04-1.30)	0.15	0.006
Body mass index (kg/m2)			
Model 1^a^	1.30 (1.20-1.42)	0.26	< 0.001
Model 2^b^	1.31 (1.20-1.42)	0.26	< 0.001
Model 3^c^	1.36 (1.19-1.56)	0.30	< 0.001
Model 4^d^	1.29 (1.05-1.58)	0.25	0.01
Waist circumference (cm)			
Model 1^a^	2.95 (1.02-8.55)	1.08	0.045
Model 2^b^	2.98 (0.99-8.93)	1.09	0.050
Model 3^c^	3.74 (0.78-17.85)	1.32	0.09
Model 4^d^	56.34 (0.001- 5.977E)	4.03	0.98
Hip circumference (cm)			
Model 1^a^	1.44 (1.30-1.60)	0.37	< 0.001
Model 2^b^	1.45 (1.31-1.61)	0.37	< 0.001
Model 3^c^	1.37 (1.22-1.55)	0.32	< 0.001
Model 4^d^	1.50 (1.31- 2.00)	0.40	0.005

* *P <0.05* statistically significant according to multivariate logistic regression;

a. Model 1: unadjusted;

b. Model 2: adjusted for age and gender;

c. Model 3: adjusted for age, gender, education, and physical activity;

d. Model 4: adjusted for age, gender, education, physical activity, and medications.

### Correlation of new anthropometric indices with metabolic parameters in the T2DM group

FBG was significantly positively correlated with waist-to-height ratio (r = 0.75; p < 0.001), AVI (r = 0.78; p < 0.001), LAP (r = 0.74; p < 0.001), CI (r = 0.7; p < 0.001), and BAI (r = 0.49; p < 0.001). HbA1c was significantly positively correlated with waist-to-height ratio (r = 0.74; p < 0.001), AVI (r = 0.75; p < 0.001), LAP (r = 0.73; p < 0.001), CI (r = 0.75; p < 0.001), and BAI (r = 0.46; p < 0.001). In the T2DM group, LDL-c was significantly positively correlated with waist-to-height ratio (r = 0.15; p = 0.02), AVI (r = 0.14; p = 0.02) and LAP (r = 0.24; p < 0.001). LDL-c/HDL-c was significantly positively correlated with waist-to-height ratio (r = 0.22; p = 0.001), AVI (r = 0.21, p = 0.001), LAP (r = 0.29; p < 0.001), CI (r = 0.15; p = 0.02) and BAI (r = 0.14, p = 0.03). HDL-c was significantly negatively correlated with waist-to-height ratio (r = -0.23; p < 0.001), AVI (r = -0.22, p = 0.001), LAP (r = -0.24, p < 0.001), and CI (r = -0.18, p = 0.005). The AIP was significantly positively correlated with LAP (r = 0.42, p < 0.001). Also, triglycerides (r = 0.36; p < 0.001), total cholesterol (TC) (r = 0.16; p = 0.01), and VLDL-c (r = 0.36; p < 0.001) were significantly positively correlated with LAP. In addition, BUN was significantly positively correlated with waist-to-height ratio (r = 0.16; p = 0.01) and AVI (r = 0.14; p = 0.03). Creatinine was significantly positively correlated with waist-to-height ratio (r = 0.17; p = 0.01), AVI (r = 0.20; p = 0.002), LAP (r = 0.14; p < 0.001) and CI (r = 0.14; p =0.03). Albuminuria was significantly positively correlated with waist-to-height ratio (r = 0.48; p = < 0.001), AVI (r = 0.50; p < 0.001), LAP (r = 0.44; p < 0.001), CI (r = 0.49; p < 0.001), and BAI (r = 0.35; p < 0.001). MAP was significantly positively correlated with waist-to-height ratio (r = 0.25; p = < 0.001), AVI (r = 0.24; p < 0.001), LAP (r = 0.20; p = 0.033), CI (r = 0.20; p = 0.002) and BAI (r = 0.25; p = 0.001). PP was positively correlated with waist-to-height ratio (r = 0.18; p = 0.005), AVI (r = 0.16; p = 0.01), LAP (r = 0.15, p = 0.01), CI (r = 0.14, p = 0.03), and BAI (r = 0.17, p = 0.01). SBP was positively correlated with waist-to-height ratio (r = 0.27; p = < 0.001), AVI (r = 0.26; p < 0.001), LAP (r = 0.22; p = 0.001), CI (r = 0.22; p = 0.001), and BAI (r = 0.25; p < 0.001). DBP was positively correlated with waist-to-height ratio (r = 0.17; p = 0.009), AVI (r = 0.18; p = 0.007), LAP (r = 0.13; p = 0.03), CI (r = 0.14; p = 0.04), and BAI (r = 0.15; p = 0.01). No significant correlations were found between ABSI and metabolic factors (Table 3).

**Table 3:** Relationships between new anthropometric indices and metabolic factors in T2DM patients (case group).

Variables	Waist/height R *P- value* *		A body shape index R P- value*		Abdominal volume index R P- value*		Conicity index R P- value*		Lipid accumulation product R P- value*		Body adiposity index R P- value*	
Fasting blood glucose (mg/dL)	0.75	< 0.001	0.05	0.41	0.78	< 0.001	0.70	< 0.001	0.74	< 0.001	0.49	< 0.001
Hemoglobin A1c %	0.74	< 0.001	0.06	0.33	0.75	< 0.001	0.75	< 0.001	0.73	< 0.001	0.46	< 0.001
Low-density lipoprotein cholesterol (mg/dl)	0.15	0.02	-0.1	0.13	0.14	0.02	0.08	0.21	0.24	< 0.001	0.12	0.059
High-density lipoprotein cholesterol (mg/dl)	-0.23	< 0.001	0.07	0.28	-0.22	0.001	-0.18	0.005	-0.24	< 0.001	-0.09	0.14
Triglycerides (mg/dL)	-0.06	0.31	0.002	0.97	-0.04	0.46	-0.06	0.33	0.36	< 0.001	-0.08	0.20
Total cholesterol (mg/dl)	-0.06	0.35	-0.08	0.2	-0.05	0.42	-0.1	0.13	0.16	0.01	-0.07	0.30
Low-density-to-high-density lipoprotein cholesterol	0.22	0.001	-0.09	0.15	0.21	0.001	0.15	0.02	0.29	< 0.001	0.14	0.03
Very low-density lipoprotein cholesterol (mg/dl)	-0.06	0.31	0.002	0.97	-0.04	0.46	-0.06	0.33	0.36	< 0.001	-0.08	0.20
Atherogenic index of plasma	0.12	0.07	-0.04	0.52	0.12	0.057	0.09	0.18	0.42	< 0.001	0.01	0.84
Blood urea nitrogen (mg/dL)	0.16	0.01	-0.05	0.42	0.14	0.03	0.11	0.07	0.1	0.12	0.07	0.24
Creatinine (mg/dL)	0.17	0.01	-0.01	0.8	0.20	0.002	0.14	0.03	0.23	< 0.001	0.08	0.19
Albuminuria (mg/24h)	0.48	< 0.001	0.01	0.87	0.50	< 0.001	0.44	< 0.001	0.44	< 0.001	0.35	< 0.001
Estimated glomerular filtration rate (mL/min/1.73m2)	-0.01	0.83	0.1	0.12	-0.05	0.44	0.02	0.67	-0.02	0.74	0.08	0.23
Systolic blood pressure (mmHg)	0.27	< 0.001	-0.05	0.45	0.26	< 0.001	0.22	0.001	0.22	0.001	0.25	< 0.001
Diastolic blood pressure (mmHg)	0.17	0.009	-0.1	0.78	0.18	0.007	0.14	0.03	0.13	0.04	0.15	0.01
Mean arterial pressure (mmHg)	0.25	< 0.001	-0.03	0.58	0.24	< 0.001	0.2	0.002	0.2	0.003	0.22	0.001
Pulse pressure (mmHg)	0.18	0.005	-0.04	0.5	0.16	0.01	0.14	0.03	0.15	0.01	0.17	0.01

Values are expressed as means ± SD. *P <0*.*05* was considered significant;

* *P <0*.*05* was considered as significant using the Pearson test for correlation between variables. R was considered as correlation coefficient.

### New and old anthropometric indices and the risk of Metabolic Syndrome (MetS)

Seventy-seven out of 110 T2DM patients (70%) had MetS based on the IDF criteria for the Iranian population, whereas there was no MetS in the healthy group (p < 0.001) (Table 1). The OR for MetS increased significantly with AVI (OR: 1.90, 95% CI: 1.57-2.29, p < 0.001), LAP (OR: 1.19, 95% CI: 1.13-1.27, p < 0.001), and BAI (OR: 1.19, 95% CI: 1.12-1.26, p < 0.001). Also, after adjusting for confounding factors (sex, age, physical activity, education, and medications), the results did not change in terms of significance (p < 0.05). Furthermore, the OR for MetS increased significantly with weight (OR: 1.05, 95% CI: 1.02, 1.08, p < 0.001), BMI (OR: 1.14, 95% CI: 1.06, 1.23, p < 0.001), WC (OR: 1.28, 95% CI: 1.19, 1.38, p < 0.001), and HC (OR: 1.19, 95% CI: 1.13, 1.25, p < 0.001). Also, after adjusting for confounding factors (sex, age, physical activity, education, and medications), the results reminded significant for WC (OR: 1.29, 95% CI: 1.18, 1.42, p < 0.001), and HC (OR: 1.07, 95% CI: 1.01, 1.14, p = 0.09) (Table 4).

**Table 4:** Odds ratios (95% CI) for metabolic syndrome according to new and old anthropometric indices.

**Variable**	**Or (CI)**	**B**	* ***P- value***
Body adiposity index (Kg/m^2^)			
Model 1a	1.19 (1.12-1.26)	0.17	< 0.001
Model 2b	1.19 (1.12-1.27)	0.17	< 0.001
Model 3c Model 4d	1.12 (1.05-1.19) 1.09 (1.01-1.17)	0.11 0.08	< 0.001 0.01
Lipid accumulation product Model 1^a^	1.19 (1.13-1.27)	0.18	< 0.001
Model 2^b^ Model 3^'^	1.20 (1.13-1.28)	0.18	< 0.001
	1.19 (1.12-1.27)	0.17	< 0.001
Model 4^d^	1.21 (1.06-1.37)	0.19	0.003
Abdominal volume index			
Model 1^a^	1.90 (1.57-2.29)	0.64	< 0.001
Model 2^b^	1.94 (1.60-2.35)	0.66	< 0.001
Model 3^c^	1.94 (1.57-2.40)	0.66	< 0.001
Model 4^d^	1.97 (1.58-2.46)	0.68	< 0.001
Weight			
Model 1^a^	1.05 (1.02, 1.08)	0.05	< 0.001
Model 2^b^	1.05 (1.02, 1.08)	0.05	< 0.001
Model 3^c^	1.01 (0.98, 1.05)	0.01	0.23
Model 4^d^	1.00 (0.96, 1.04)	0.002	0.90
Body mass index (kg/m^2^)			
Model 1^a^	1.14 (1.06, 1.23)	0.13	< 0.001
Model 2^b^	1.14 (1.06, 1.23)	0.13	< 0.001
Model 3^c^	1.06 (0.98, 1.16)	0.06	0.11
Model 4^d^	0.99 (0.89, 1.10)	-0.008	0.87
Waist circumference (cm)			
Model 1^a^	1.28 (1.19, 1.38)	0.25	< 0.001
Model 2^b^	1.29 (1.19, 1.40)	0.25	< 0.001
Model 3^c^	1.29 (1.19, 1.41)	0.26	< 0.001
Model 4^d^	1.29 (1.18, 1.42)	0.26	< 0.001
Hip circumference (cm) Model 1^a^	1.19 (1.13, 1.25)	0.17	< 0.001
Model 2^b^	1.20 (1.14, 1.26)	0.18	< 0.001
Model 3^c^	1.13 (1.07, 1.19)	0.12	< 0.001
Model 4^d^	1.07 (1.01, 1.14)	0.07	0.009

* *P < 0.05* statistically significant according to multivariate logistic regression;

a. Model 1: unadjusted;

b. Model 2: adjusted for age and gender;

c. Model 3: adjusted for age, gender, education, and physical activity;

d. Model 4: adjusted for age, gender, education, physical activity, and medications.

## DISCUSSION

This study evaluated the relationship between new and old anthropometric indices and the risk of T2DM and its cardiometabolic complications. All anthropometric indices except ABSI were greater in patients with T2DM compared with the participants in the control group. AVI, LAP, and CI were strongly and BAI was moderately associated with FBS and HbA1c. These indices showed similar correlations with SBP, DBP, PP and MAP. Also, new anthropometric indices have predictive ability for risk of T2DM and MetS, similar to BMI. LAP, and BAI have equal chance of predicting T2DM in our study. Obesity as measured by these indices is more related to T2DM because abdominal obesity has been described as a stronger independent predictor, not only of cardiovascular events and mortality, but also of T2DM.

Regarding the ABSI, there was no significant difference between the two groups. Additionally, the ABSI did not significantly correlate with any components of the MetS. Similarly, Gomez et al and Hardy et al., reported no relationship between ABSI and metabolic parameters.^24^^,^ 25 The ABSI was developed by Krakauer et al. who normalized WC to BMI and height.^26^ Although ABSI was significantly correlated with age and WC in the Gomez-Peralta et al., study, it seems not to be effective in relation to metabolic diseases.^24^

In this study, the AVI was significantly correlated with glucose, lipid profile, blood pressure, and renal factors. The AVI formula is based on WC and WHR. In line with our findings, Guerrero-Romero et al. found that AVI was strongly related to impaired glucose tolerance (IGT) and T2DM.^27^ It also showed the highest OR values in their study, which is not consistent with our findings.^27^ Although the OR for MetS increased significantly with AVI, the OR for T2DM did not attain significance. In another study, Ehrampoush et al., evaluated the relationship between anthropometric indices and body fat content. They suggested that AVI is more accurate than the other indices for evaluating deposition of fat in the abdominal area and body fat percentage.^28^ More studies with larger sample sizes are required to confirm this.

In the present study, LAP was significantly associated with blood glucose, lipid profile, SBP, and DBP. Similarly, in the study by Angelo Vieira et al., which evaluated the correlation between LAP Index and cardiovascular risk factors in hospitalized persons, LAP was significantly correlated with TC, HDL-c, FBS, and SBP.^29^ Additionally, other researchers found correlations between the LAP index and FBS, HDL-c, and SBP in both healthy subjects and patients with higher cardiovascular risk.^30^ None of these studies found significant correlations between the LAP index and DBP or LDL. The LAP index also increased the risk of T2DM by 30%. Prior studies revealed strong predictive accuracy for MetS.^9^ Overall, the LAP showed stronger relationships with metabolic factors than the other adiposity measures. The LAP index should be recognized as a parameter calculated based on WC and serum triglyceride levels that is strongly correlated with visceral fat.

In our study, CI had significant correlations with FBS, HbA1c, LDL-c/HDL-c, HDL-c, albuminuria, SBP, and DBP. Likewise, in the study by Ghosh et al., CI was associated with high blood glucose.^31^ Other studies also reported that CI was associated with triglycerides and TC.^27^ Variables such as weight, height, and abdominal circumference are used to estimate the CI. However, this study suggested that the CI could not predict risk of T2DM. In contrast, the Andrade et al. study identified the CI as tool for estimating the risk of DM. Our smaller sample size might have contributed to this result.^32^

In the present study; the BAI had significant correlations with FBS, HbA1c, LDL-c/HDL-c, HDL-c, SBP, and DBP. The BAI was developed by Bergman et al., based on HC and height.^33^ It increased the risk of T2DM up to 20%, even after adjusting for confounding variables such as age, sex, and medications. Similarly, in the study by Oliveira et al., the BAI represented an increase of 8.4% in the risk of a patient developing T2DM.^34^ Corroborating the findings of Bergman et al. and Lopez et al., our findings confirmed this relationship, highlighting the BAI as an effective parameter in T2DM.^33^^,^ 35

However, limitations of the present study include not assessing dietary intakes, the small sample size, and the fact that the case-control study design does not allow for causal inference.

In conclusion, among old and new anthropometric indices, BMI, LAP, and BAI were found to be most associated with T2DM and its related complications. HC and AVI were also found to be sensitive markers. ABSI was not a sensitive marker. So, some of these indices have notable clinical importance for evaluation of T2DM control and could be used in clinical practice selectively.

Abbreviations: BMI; body mass index, CI; conicity index, WC; waist circumference, HC; hip circumference, WHR; waist-to-hip ratio, SBP; systolic blood pressure, DBP; diastolic blood pressure, FBG; fasting blood glucose, HbA1C; glycosylated hemoglobin, TG; triglyceride, CHOL; total cholesterol, HDL; high-density lipoprotein, LDL; low-density lipoprotein cholesterol, VLDL; very low-density lipoprotein, eGFR; estimated glomerular filtration rate, BUN; blood urea nitrogen, Cr; creatinine, MAP= mean arterial pressure; PP = pulse pressure; AIP= atherogenic index of plasma; ABSI = a body shape index; AVI = abdominal volume index; BAI = body adiposity index; LAP = lipid accumulation product; MetS = metabolic syndrome.
